# Pluripotent stem cell based and crispr engineered models of neurodegeneration to assess neuronal networks

**DOI:** 10.1002/alz.087335

**Published:** 2025-01-03

**Authors:** Henrik Ahlenius

**Affiliations:** ^1^ Stem Cells, Aging and Neurodegeneration, Lund University, Lund, Lund Sweden

## Abstract

**Background:**

Stem cell based models of neurodegeneration are emerging as valuable tools to study neuronal networks as well as for drug discovery and testing. Drugs identified using stem cell based models are now entering clinical trials.

**Method:**

We have generated *CHMP2B, APP, PSEN* and *Tau*‐mutated and transgenic human embryonic and induced pluripotent stem cell lines, using CRISPR genome editing, with the purpose to create human *in vitro* disease models. To date, most studies have focused on neuronal alterations; however, we have developed co‐culture systems in which neurons and glia are independently generated from human pluripotent stem cells and combined *in vitro*. We are now using these models to study neuronal networks and the involvement of astrocytes in FTD and AD.

**Result:**

With this approach, we have identified alterations in the endolysosomal system of FTD astrocytes, a higher capacity of astrocytes to uptake and respond to glutamate, and neuronal network hyperactivity as well as excessive synchronization. Overall, our data indicates that astrocyte alterations precede neuronal impairments and could potentially trigger neuronal network changes, indicating the important and specific role of astrocytes in disease development.

**Conclusion:**

Here we present our improved and updated strategy and results of combining pluripotent stem cells, CRISPR genome engineering and forward programming to generate complex co‐culture models to study neuronal network alterations in cardinal neurodegenerative disorders.

